# Ultrasound-accelerated thrombolysis in high-risk perioperative pulmonary embolism: two case reports and review of literature

**DOI:** 10.1186/s13741-021-00205-4

**Published:** 2021-10-18

**Authors:** Götz Schmidt, Fabian Edinger, Christian Koch, Matthias Wolff, Christoph Biehl, Rüdiger Hörbelt, Michael Sander

**Affiliations:** 1 Department of Anaesthesiology, Operative Intensive Care Medicine and Pain Therapy, Justus Liebig University of Giessen, Rudolf-Buchheim-Strasse 7, 35392 Giessen, Germany; 2 Department of Trauma, Hand and Reconstructive Surgery, Justus Liebig University of Giessen, Rudolf-Buchheim-Strasse 7, 35392 Giessen, Germany; 3 Department of General and Thoracic Surgery, Justus Liebig University of Giessen, Rudolf-Buchheim-Strasse 7, 35392 Giessen, Germany

**Keywords:** Postoperative, Surgical patients, Surgery, Embolectomy

## Abstract

**Introduction:**

Treatment of high-risk pulmonary embolism (PE) in perioperative patients remains challenging. Systemic thrombolysis is associated with a high risk of major bleedings and intracranial haemorrhage. High mortality rates are reported for open pulmonary embolectomy. Therefore, postoperative surgical patients may benefit substantially from catheter-directed ultrasound-accelerated thrombolysis (USAT).

**Case presentation:**

We report two cases of high-risk perioperative PE. Both patients developed severe haemodynamic instability leading to cardiac arrest. After the implantation of a veno-arterial extracorporeal membrane oxygenation (ECMO), they were both successfully treated with USAT. Adequate improvement of right ventricular function was achieved; thus, ECMO could be successfully weaned after 3 and 4 days, respectively. Both patients showed favourable outcomes and could be discharged to rehabilitation.

**Conclusion:**

Current guidelines on treatment of PE offer no specific therapies for perioperative patients with high-risk PE. However, systemic thrombolysis is often excluded due to the perioperative setting and the risk of major bleeding. Catheter-directed thrombolysis was shown to utilise less thrombolytic agent while obtaining comparable thrombolytic effects. The risk for major bleeding (including intracranial haemorrhage) is also significantly lowered. Until further trials determining the value of adopted treatment strategies of high-risk PE in perioperative patients are available, USAT should be considered in similar cases.

## Background

Pulmonary embolism (PE) is a well-known clinical condition and may be related to increased morbidity and mortality. The Virchow’s triad, consisting of stasis, endothelial injury and hypercoagulability, is fulfilled by different predisposing factors, which can be classified into patient- and setting-related risk factors (Rogers et al. 2012; Anderson and Spencer 2003; Konstantinides et al. 2020); setting-related risk factors are usually temporary and include not only fractures of the lower limb, hip or knee replacement, major trauma and blood transfusion, but also immobility and history of, and deep venous thrombosis (DVT) itself. These are of enormous importance in the perioperative environment, and often, many of them often occur simultaneously. PE may lead to an obstructive shock with reduced RV function and consecutive reduced left-ventricular output resulting in sudden haemodynamic instability and cardiac arrest (Konstantinides et al. 2020). RV failure and haemodynamic instability both increase the early postoperative mortality (Harjola et al. 2016).

The pulmonary embolism severity index (PESI) (Aujesky et al. 2005) has been developed to assess the mortality risk in patients with PE. The predicted mortality, which is lowest in class I (0–1.6%) and highest in class V (10–24.5%) may lead to an appropriate, individual therapy. To maintain haemodynamic and respiratory stability, oxygen supply and non-invasive or invasive mechanical ventilation may be necessary. RV failure and subsequent systemic hypotension should be treated with volume optimisation, vasopressors and inotropes. Especially after high-risk PE with cardiac arrest, veno-arterial extracorporeal membrane oxygenation (VA-ECMO) may be used to achieve haemodynamic stability and to guarantee adequate tissue oxygenation (Yusuff et al. 2015). To prevent further thrombus formation, all patients should receive therapeutic anticoagulation.

However, particularly in high-risk PE, additional causal therapy is needed; therefore, different reperfusion strategies exist. Systemic thrombolysis may be achieved by using fibrinolytic agents (Konstantinides et al. 2020). Surgical pulmonary embolectomy is performed via an open access to the two main pulmonary arteries and the consecutive removal of obstructive clots (Konstantinides et al. 2020). In addition to systemic thrombolysis and surgical revascularisation, percutaneous catheter-directed therapy may also be considered as second-line therapy (Konstantinides et al. 2020). Via the femoral or jugular vein, a catheter is brought to the pulmonary artery where aspiration thrombectomy or local low-dose thrombolysis is performed (Piazza et al. 2015a). Furthermore, ultrasound-assisted thrombolysis devices deliver ultrasound to ensure an increase in the clots’ surface area and therefore improve the effect of the low-dose thrombolytic agent (Braaten et al. 1997).

The management of perioperative patients with high-risk PE is challenging. According to current ESC, AHA and CHEST guidelines, major surgery is a relative exclusion criterion for systemic thrombolysis due to the high risk of major bleeding complications (Konstantinides et al. 2020; Jaff et al. 2011; Kearon et al. 2016). Surgical embolectomy must also be considered with caution, as some studies report rather high mortality rates. The percutaneous catheter-directed approach may be an attractive option in haemodynamically unstable patients suffering from PE after major surgery. We report the first treatment—to our knowledge—of perioperative high-risk PE leading to cardiac arrest and VA-ECMO implantation with ultrasound-accelerated catheter-directed thrombolysis (USAT).

### Case 1

**Fig. 1:**
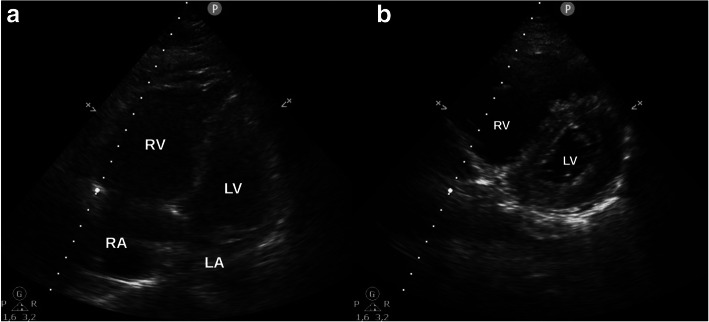
**a** Apical four-chamber view showed severe RV dilatation and **b** parasternal short axis view revealed flattened interventricular septum (LA = left atrium, RA = right atrium, LV = left ventricle, RV = right ventricle)

**Fig. 2:**
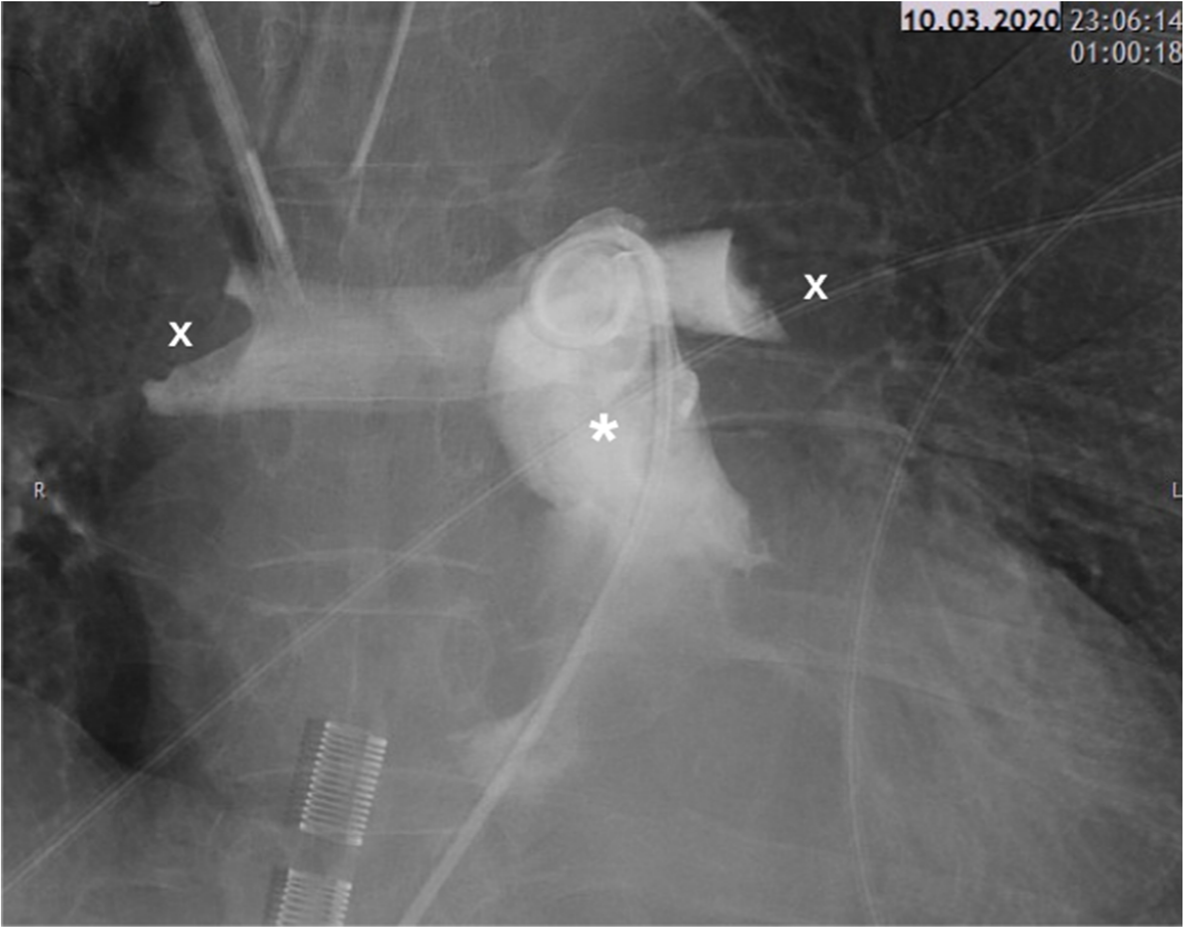
Angiography showed bilateral occlusion of both main pulmonary arteries (* = pulmonary trunk, x = thrombotic burden)

A 59-year-old female with a body mass index (BMI) of 28.4 kg/m^2^ underwent elective cemented knee replacement surgery. With a medical history of arterial hypertension, intraoperative analgesia was achieved with spinal anaesthesia (3.4 ml of bupivacaine 0.5%) and sedation with propofol (2.4 mg/kg/h). During the first three postoperative days (POD), her recovery proceeded without any abnormalities. Prophylactic anticoagulation was achieved with weight-adjusted low-molecular weight heparin (LMWH, certoparin 3000 I.E. s.c. per day). On POD 4, the patient developed dyspnoea during mobilisation. The subsequent CT scan revealed bilateral PE; therefore, the patient was transferred to the intermediate care unit. On admission, the patient was awake, responsive and did not present dyspnoea in supine position. Anticoagulation was performed with a bolus of 5000 I.E. of heparin, followed by enoxaparin (2× 0.8 mg s.c. per day). In good overall condition and with a corresponding PESI-Score of 59 (class I, mortality risk 0–1.6%), the patient was readmitted to the general ward in the morning of POD 5. In the evening, the patient collapsed again and presented with dyspnoea and tachycardia. Transthoracic echocardiography revealed severe RV dilatation (Fig. 1a), tricuspid regurgitation, decreased tricuspid annular plane systolic excursion (TAPSE, 11 mm) and flattened interventricular septum (Fig. 1b). Due to acute cardiac decompensation and pulseless ventricular tachycardia, cardiopulmonary resuscitation (CPR) and intubation had to be performed. Haemodynamic stabilisation could only be achieved after implantation of a VA-ECMO. Noradrenaline was given with a maximum rate of 0.06 μg/kg/min. Levosimendan (0.1 μg/kg/min) ensured additional inotropic support. The subsequent angiography showed bilateral occlusion of the main pulmonary arteries (Fig. 2).

**Fig. 3:**
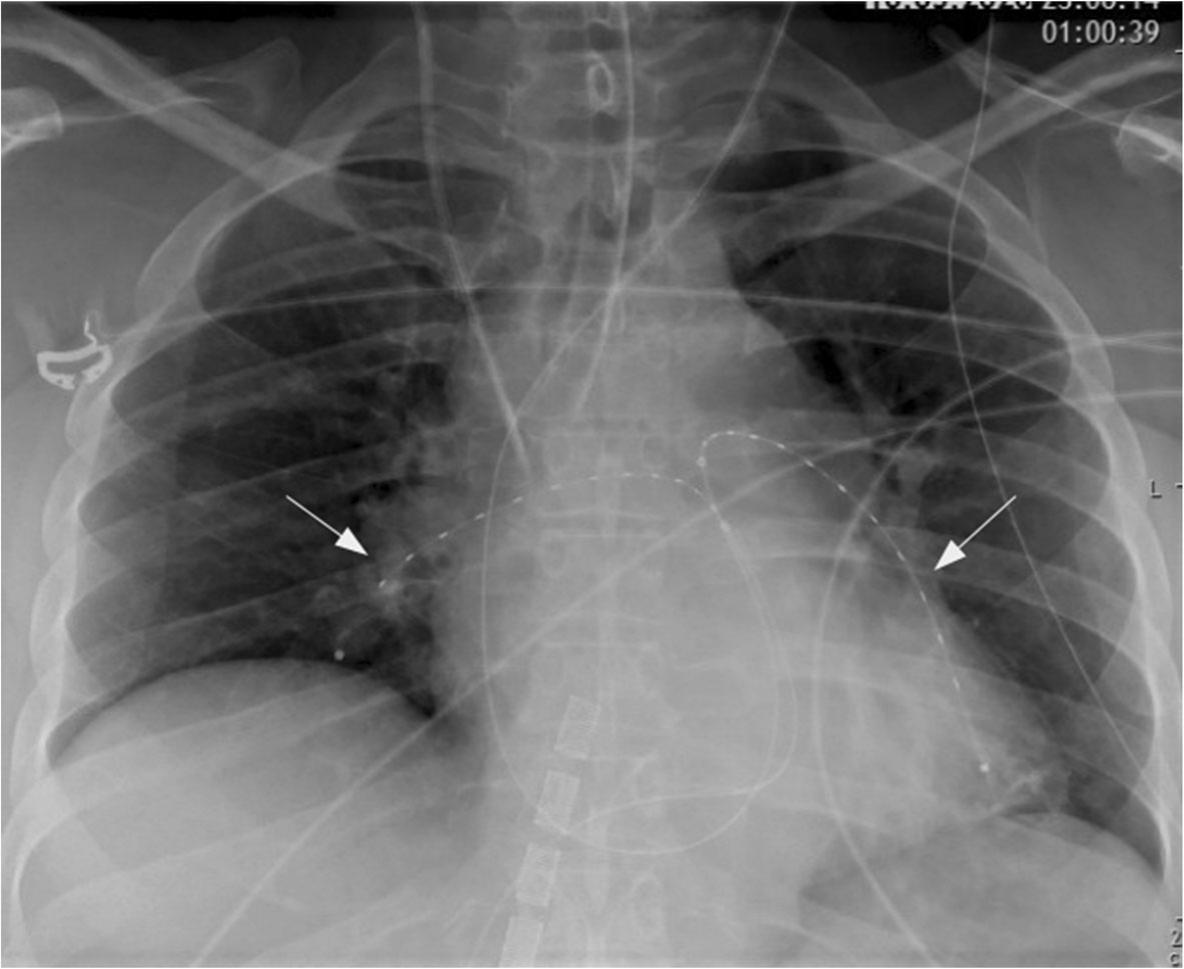
Chest X-ray shows both catheters in situ (arrows)

**Fig. 4:**
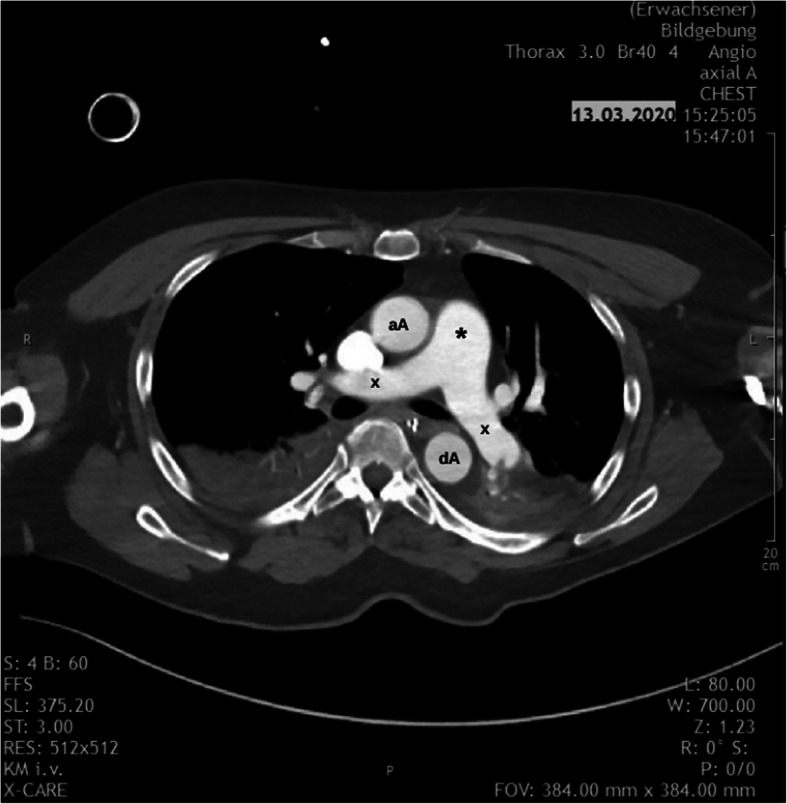
CT scan revealed regredient embolic burden (x) and improved recanalization after ECMO explantation (* = pulmonary trunk, aA = ascending aorta, dA = descending aorta)

According to the PESI-Score, the patient was now classified in class V (very high mortality risk, 10–24.5%), underlining the need for urgent reperfusion therapy. However, according to the guidelines, systemic thrombolysis was relatively contraindicated due to the high bleeding risk after major surgery (Konstantinides et al. 2020; Jaff et al. 2011; Kearon et al. 2016). As a consequence of the interdisciplinary team discussion, ultrasound-accelerated catheter-directed thrombolysis (EkoSonic Endovascular System [EKOS Corporation, a Boston Scientific Company, Bothell, WA, USA]) was performed. One catheter was inserted into the right pulmonary artery via the right femoral vein. Subsequently, another catheter was brought to the left pulmonary artery via the left jugular vein (Fig. 3). After the ultrasound emission, each catheter released 1 mg/h rtPA for the first 5 h, followed by 0.5 mg/h rtPA for 10 h. Therapeutic anticoagulation was then established with argatroban (0.5 μg/kg/min), resulting in a partial thromboplastin time (aPTT) of 60–70 s. Due to the stabilisation of RV-function, ECMO support was ended 3 days after the implantation. An additional CT scan revealed regression of the thrombotic burden (Fig. 4), revealing only small clots in the lobar arteries. Due to respiratory and haemodynamic stabilisation, the patient was extubated 1 day later. Subsequently, the therapeutic anticoagulation regimen was changed to oral treatment with apixaban (2× 10 mg p.o. for 1 week, 2× 5 mg p.o. subsequently). Ten days later, the patient was transferred to the general ward, from which she was discharged to ambulatory treatment in good condition after 9 days.

### Case 2

A 59-year-old female with a BMI of 23.5 kg/m^2^ suffering from renal cell carcinoma (RCC) metastasis in the right lobe of the liver, underwent elective right hemihepatectomy. The patient’s medical history included RCC treated with nephrectomy, splenectomy and pancreatic left resection in 2003. Currently, the patient presented in a good condition. After the uneventful surgery, during which 84% of the liver was resected, the patient was transferred to the intensive care unit. Intermittent pneumatic compression (Kendall SCD 700, Cardinal Health, Ireland) was used as deep-venous thrombosis prophylaxis. Prophylactic anticoagulation was first achieved with heparin (400 I.E./h) and was subsequently changed to certoparin (3000 I.E. s.c. per day). Her recovery proceeded without any abnormalities; thus, she was discharged to the general ward at POD 1. One day later, she collapsed during mobilisation and was immediately transferred to the radiology department. On the way, she became haemodynamically unstable and CPR was started. Orotracheal intubation was performed and the patient was transferred to the intensive care unit, where a VA-ECMO was implanted. After haemodynamic stabilisation, a CT scan was performed and revealed massive bilateral PE spreading through all lobar and segmental arteries. Due to the large thrombotic burden and the massive derangement of coagulation (prothrombin time < 7% of the norm, international normalised ratio > 6.1, aPTT > 120 s), an interdisciplinary decision was made to pursue interventional treatment. Pulmonary angiography and consecutive catheter implantation (EkoSonic Endovascular System [EKOS Corporation, a Boston Scientific Company, Bothell, WA, USA]) in each pulmonary artery allowed the performance of USAT. The left pulmonary artery catheter was inserted through the left femoral vein, and the right pulmonary artery was reached through the right jugular vein. During the following 12 h, 12 mg of rtPA were administered (0.5 mg/h/catheter). After 6 h, an additional CT scan already revealed partial recanalisation of the lobar arteries. Noradrenaline was administrated at a maximum dose of 0.19 μg/kg/min and inotropic support was delivered using low-dose dobutamine (3 μg/kg/min). During USAT with rtPA and therapeutic anticoagulation with argatroban (at a maximum of 0.46 μg/kg/min, resulting in an aPTT of 60–80 s), a major abdominal bleed from the operating area occurred. The bleeding required massive transfusion and had to be treated in the operating theatre twice. Subsequently, examinations of RV function were performed with transoesophageal echocardiography (TEE). Daily improvements in biventricular function were recognised; as a result, the ECMO was removed after 4 days. Subsequently, the patient underwent prolonged intensive care treatment, including tracheotomy, dialysis and complicated weaning. Empyema of the lower lobe due to a lung infarction-associated pneumonia was treated surgically before the patient’s condition improved. She was transferred to the general ward 41 days after admission to the intensive care unit and was discharged to rehabilitation 2 weeks later. Today, the patient is independent in daily life and in good neurological and stable general condition.

## Discussion

We report the first to two cases to our knowledge of perioperative high-risk PE including cardiac arrest that were treated by ultrasound-accelerated catheter-directed thrombolysis (USAT). Despite CPR, both patients’ initial haemodynamic stabilisation was achieved by VA-ECMO therapy. Subsequently, successful thrombolysis with USAT led to favourable outcomes. Treatment of high-risk PE remains challenging. According to the current guidelines on Acute Pulmonary Embolism, patients with high-risk PE should receive therapeutic anticoagulation and systemic thrombolytic therapy (ESC class I recommendation, CHEST class II recommendation) (Konstantinides et al. 2020; Kearon et al. 2016). Supplementary, the AHA guidelines notes systemic thrombolysis is only reasonable for patients with acute PE and acceptable risk of bleeding complications (class IIa recommendation) (Jaff et al. 2011). However, major surgery is a relative exclusion criterion for systemic thrombolysis (Konstantinides et al. 2020; Jaff et al. 2011; Kearon et al. 2016). The risk of bleeding complications—up to a 9.9% rate of severe bleedings and a 1.7–5.0% rate of intracranial haemorrhage—is not negligible and must be considered carefully (Fiumara et al. 2006; Marti et al. 2015). If systemic thrombolysis is contraindicated or has failed, surgical embolectomy should be performed (ESC class I recommendation, AHA class IIa recommendation) (Konstantinides et al. 2020; Jaff et al. 2011). Surgical thrombectomy is a highly invasive procedure where cardioplegic cardiac arrest with cardiopulmonary bypass is usually necessary. In comparison to systemic thrombolysis, Lee et al. reported similar survival rates and a lower risk of stroke (Ruohoniemi et al. 2018). However, surgical embolectomy showed the highest mortality rates (32%) after cardiac arrest (Wu et al. 2013). It must be noticed, according to CHEST guidelines, surgical embolectomy should only be considered in the context of chronic thromboembolic pulmonary hypertension. Percutaneous catheter-directed thrombolysis may be considered as second-line therapy (class IIa recommendation) (Konstantinides et al. 2020; Jaff et al. 2011; Kearon et al. 2016). Compared to systemic thrombolysis, currently available USAT devices require only 10% of the thrombolytic agent (Ruohoniemi et al. 2018). USAT has been reported to be safe in non-surgical patients. Kuo et al. reported a significant improvement in mean pulmonary artery pressure and echocardiography after acute PE treated with catheter-directed therapy, with clinical success achieved in 85.7% of patients (Kuo et al. 2015). Tapson et al. showed an improved right ventricular-to-left ventricular diameter ratio and reduced embolic burden even when low-dose rtPA (8–24 mg) was administered (Tapson et al. 2018). Nevertheless, a 4% rate of major bleedings and one intracranial haemorrhage that was attributed to rtPA occurred. These encouraging results could be confirmed by Piazza et al., who reported only one severe bleeding among 150 patients (Piazza et al. 2015b). A 10% rate of moderate bleeding was observed after the cumulative admission of 24 mg rtPA. Furthermore, no intracranial haemorrhage occurred. Kucher et al. performed a randomised controlled trial comparing USAT therapy with heparin only (Kucher et al. 2014). No major bleeding occurred in the intervention group after the admission of 10–20 mg rtPA. A higher overall risk of bleeding could not be detected. Another meta-analysis confirmed that, compared to systemic thrombolysis, USAT results in a lower risk of major bleedings (Kaymaz et al. 2017). Although USAT improved haemodynamic parameters, it failed to reduce mortality in intermediate-risk PE patients. Thus, the benefit of USAT is questionable in this scenario and must be weighed against the risks and possible procedural complications.

Unfortunately, the current guidelines do not offer any specific strategies concerning the perioperative setting. It must be highlighted that treatment options are strongly limited by the high bleeding risk during the perioperative period. Moreover, there are only few reports regarding the treatment of perioperative high-risk PE. Some patients were solely treated with therapeutic anticoagulation and did not receive any further thrombolytic therapy (Smith and Murauski 2017; Mao et al. 2017). Smith et al. reported a case of a perioperative submassive high-risk PE in the right upper lobe after hip hemiarthroplasty leading to cardiac arrest (Smith and Murauski 2017). Return of spontaneous circulation was achieved after 2 min. Further therapy with anticoagulation and inotropic support with dobutamine led to adequate stabilisation; thus, thrombolytic therapy was withdrawn due to the high risk of bleeding. Hartmannsgruber et al. reported the successful application of systemic thrombolysis after high-risk perioperative PE in a patient with morbid obesity (Hartmannsgruber et al. 1996). However, the patient had undergone umbilical hernia repair with a very low bleeding risk. Another patient undergoing major trauma surgery was treated with USAT after intraoperative massive high-risk PE (Dudaryk et al. 2018). Haemodynamic stabilisation was achieved with phenylephrine and fluid therapy. In contrast, both of our cases presented with cardiac arrest. Haemodynamic stabilisation could only be achieved by VA-ECMO underlining the immediate need for a reperfusion therapy that decreases mortality (Marti et al. 2015).

In our cases, an interdisciplinary team discussion involving the initial surgeons, cardiothoracic surgeons, cardiologists, radiologists and intensive care physicians resulted in the decision to perform USAT. The concept of a multidisciplinary team for the management of high-risk PE is recommended by the 2019 ESC Guidelines for the management of acute PE (Konstantinides et al. 2020). Due to the haemodynamic instability requiring ECMO, the need for revascularisation therapy—despite therapeutic anticoagulation—was obvious. Therefore, our team carefully weighed the above-mentioned characteristics of the three revascularisation options. Systemic thrombolysis was withdrawn due to the high risk of fatal bleeding during the postoperative period. In case 2, cardiac arrest after liver surgery led to the massive derangement of coagulation, exponentially increasing the risk for major bleeding and intracranial haemorrhage. Conscious of the highly invasive nature of the procedure and the high mortality rates associated with surgical embolectomy after cardiac arrest, our team decided that local catheter-directed therapy was the most feasible treatment strategy. The additional use of ultrasound was thought to decrease the treatment time and dose of the thrombolytic agent, which should therefore result in decreased morbidity and mortality (Owens 2008). Compared with catheter-directed thrombolysis without ultrasound, USAT was first shown to reduce the mean thrombolysis time and the incidence of treatment-related haemorrhagic complications. Initial data also revealed that USAT achieved more complete thrombus removal utilizing lower thrombolytic doses (Graif et al. 2017; Lin et al. 2009). However, recent evidence has emerged indicating no differences in reduction of mean pulmonary artery pressure and lytic doses with or without ultrasound (Liang et al. 2016; Rao et al. 2019; Rothschild et al. 2019). Furthermore, current studies showed similar overall survival rates, ICU and hospital length-of-stays, and long-term quality of life as well (Rao et al. 2019; Rothschild et al. 2019). These findings question the additional benefits and cost effectiveness of USAT, suggesting that current evidence comparing catheter-directed thrombolysis with or without ultrasound is equivocal and further randomized controlled trials remain necessary (Avgerinos et al. 2018).

Although percutaneous catheter-directed treatment is associated with the lowest rate of major bleeding complications of all reperfusion strategies, in case 2, major bleeding of the surgical site occurred during USAT therapy. Despite the high bleeding risk after major liver surgery, the thrombolytic agent administered may have accelerated the severity of bleeding. Our first patient suffered from recurrent PE although therapeutic anticoagulation had been started only hours before. Additionally, the risk according to the PESI-score was very low. However, HIT tests revealed no pathological findings, although the test on tissue factor antibodies showed a marginally but insignificant result. As reported in our cases, further anticoagulation was achieved with argatroban. In comparison to UFH, argatroban was shown to significantly accelerate thrombolysis in vitro, despite no difference in PTT prolongation being seen (Yamada et al. 2003). With UFH, the same effect could only be seen with higher doses resulting in an over-prolonged PTT above 180 s. While both patients were treated with USAT and argatroban, a fast improvement of the pulmonary artery pressure was monitored. In summary, we report two cases of high-risk perioperative PE in which USAT in conjunction with ECMO was performed. The immediate start of high-quality resuscitation led to a closely patient-adapted therapy with minimised application of thrombolytic agents and may therefore have helped to achieve favourable neurological outcomes. However, the current guidelines for the management of acute PE do not offer any specific strategy for the treatment of perioperative patients. Until further trials evaluating the value of adopted therapies including catheter-directed thrombolysis in perioperative patients suffering from high-risk PE are available, USAT in conjunction with ECMO should be taken into due consideration in similar cases.

## Data Availability

The datasets used during the current study are available from the corresponding author on reasonable request.

## Abbreviations

aPTT: Partial thromboplastin time
BMI: Body mass index
CPR: Cardiopulmonary resuscitation
DVT: Deep venous thrombosis
ECMO: Extracorporeal membrane oxygenation
HIT: Heparin-induced thrombocythemia
LMWH: Low-molecular weight heparin
PE: Pulmonary embolism
POD: Postoperative day
RCC: Renal cell carcinoma
rtPA: Recombinant tissue-type plasminogen activator
TAPSE: Tricuspid annular plane systolic excursion
TEE: Transoesophageal echocardiography
UFH: Unfractionated heparin
USAT: Ultrasound-accelerated thrombolysis
VA-ECMO: Veno-arterial extracorporeal membrane oxygenation
